# Outcomes of pars plana vitrectomy for visually significant floaters in Northern Alberta

**DOI:** 10.1186/s40942-025-00676-3

**Published:** 2025-05-06

**Authors:** Malshi Karunatilake, Brendon Fijardo, Eugene Michael, Rizwan Somani

**Affiliations:** 1 Department of Ophthalmology and Visual Sciences, University of Alberta, Royal Alexandra Hospital, 2319 Active Treatment Centre, 10240 Kingsway Avenue NW, Edmonton, AB T5H 3V9 Canada; 2 Alberta Retina Consultants, 10924 107 Ave NW #400, Edmonton, AB T5H 0X5 Canada

**Keywords:** Vision degrading myodesopsiae, Floaters, Pars plana vitrectomy, Posterior vitreous detachment, Canada

## Abstract

**Background:**

Vision degrading mydesposiae (VDM) can have a significant impact on a patient’s quality of life. Pars plana vitrectomy (PPV) is a surgical modality used to treat a variety of vitreoretinal diseases and is an accessible treatment option for relief of VDM. This article analyzes outcomes and postoperative complications in a large, local sample of patients who have undergone PPV for symptomatic floaters from a northern Alberta perspective.

**Methods:**

A retrospective chart review was conducted at Alberta Retina Consultants in Edmonton, Alberta. Patients who underwent PPV for VDM between 2017 and 2023 were identified. Only cases with floaters due to PVD and asteroid hyalosis were included; cases of prior vitrectomies, scleral buckle surgeries as well as cases of secondary myodesopsiae (i.e. uveitis) were excluded.

**Results:**

A total 452 eyes were identified and following application of exclusion criteria, 410 eyes of 308 patients were included. There were 157 male and 151 female patients with the average age of participants being 68 years (standard deviation (SD) ± 9). Vitrectomy was performed due to symptomatic PVD in 400 eyes (98%) with 10 eyes (2%) being symptomatic due to asteroid hyalosis. There were 181 phakic eyes (44%) and 229 eyes and pseudophakic eyes (56%). There were 26 patients (25.2%) who elected to undergo floaterectomy in the contralateral eye. There was no significant difference in visual acuity noted between preoperative and postoperative periods.

**Conclusions:**

The current study supports PPV as an effective treatment option for symptomatic floaters. There was no statistically significant difference between preoperative and postoperative visual acuity. However, 29.9% of eyes underwent floaterectomy in the contralateral eye, which is in support of patient satisfaction. The observed complication rate was 7.3%, with retinal detachment being the most common complication. Of the 410 eyes, 30 eyes (7.3%) had postoperative complications which included adverse events that were recorded to have occurred between the immediate postoperative period and three years after vitrectomy. Complications include retinal detachment (2.4%), elevated IOP (1.5%), clinically significant cataract requiring surgery (1.5%), vitreous hemorrhage (0.73%), cystoid macular edema (0.98%), dislocated IOL (0.24%), endophthalmitis (0.24%) and epiretinal membrane (0.24). There was a statistically significant difference in visual acuity between preoperative and postoperative periods for eyes with complications.

## Background

Floaters, or myodesopsia, are an omnipresent vitreous entity that are visually significant for some individuals. Primary myodesopsiae are intrinsic to the posterior segment, such as muscae volitantes, which are embryological remnants from the *vasa hyaloidea propria* during development of the vitreous (Table 1). They are typically minimally symptomatic and therefore do not require removal [1]. Symptoms typically occur after a posterior vitreous detachment. This is a vitreodegenerative process whereby liquefaction (synchysis) precedes the aggregation of collagen fibrils (syneresis) leading to vitreoretinal separation and hyaloidal displacement, vitreous contraction and neomyodesopsiae [2]. Secondary myodesopsiae are extrinsic to the vitreo-hydrogel matrix and include inflammatory debris, hemorrhage, opacities or iatrogenic floaters such as silicone oil droplets [3]. Another common induction of floaters occurs following cataract surgery, as the vitreous migrates anteriorly towards the nodal point of the eye, causing symptoms. While there is no clear indication for vitreous removal, a recent posterior vitreous detachment (PVD) or cataract surgery may validate the patients’ concerns, thereby addressing their symptoms with meticulous removal of the retrolental vitreous to alleviate symptoms – however, care must be taken during anterior clearance to avoid inadvertent zonulolysis [4].

**Table 1:** Etiology of primary vitreous floaters and their descriptions

Primary myodesopsiae	Description
Vitreosenesence ex. PVD	Weiss ring, vitreous condensation
Embryological	Muscae volitantes
Hereditary disorders ex. Marfan Syndrome, Wagner Syndrome, Stickler syndrome	Vitreous veils interspersed in an optically empty vitreous
Constitutional ex. Axial Myopia	Vitreous condensation

The vitreous humor is a transparent biofluid and alternation in its composition or vitreous degeneration can lead to symptomatic floaters. Indication for surgery, known informally as the ‘floaterectomy’, is based on patient symptoms and the presence of vitreous opacities on clinical examination and/or multimodal imaging. These are referred to as vision degrading myodesopsiae (VDM), which can have a significant detrimental impact on the patient’s quality of life, visual acuity and contrast sensitivity [6–8]. The most common symptoms reported include large opacities that interfere with clear vision, particularly with reading and driving [9].

Vitrectomy is an accessible, safe and effective treatment for relief of VDM with high patient satisfaction [10]. Sebag et al. studied one hundred ninety-five phakic eyes that underwent limited vitrectomy with 25-gauge instruments for symptomatic vitreous floaters [7]. The study reports improved postoperative visual acuity (VA) and normalization of contrast sensitivity function (CSF). Complications include 3 retinal detachments that underwent successful repair, 3 retinal tears, 2 epiretinal membranes, 2 vitreous hemorrhages that spontaneously resolved and 4 recurrent floaters from new PVD which were addressed with re-operation. Mason et al. reported improved postoperative VA and only three eyes with postoperative complication, with one case of cystoid macular edema and three cases transient vitreous hemorrhage, in a sample of sixty-eight eyes [11]. Schulz-Key reports 88% patient satisfaction with floaterectomy with 4 cases of postoperative retinal detachment [12].

The purpose of this study was to analyze safety, outcomes and postoperative complications in a larger, local sample of patients that have undergone floaterectomy for symptomatic floaters. This study summarizes the findings of pars plana vitrectomy (PPV) for VDM from a single center in Alberta, Canada, with pertinence to complication rates, visual acuity outcomes and patient feedback.

## Methods

The study consists of a retrospective chart review which was conducted at Alberta Retina Consultants, Edmonton, Alberta. It serves as a single tertiary referral center with surgical procedures performed exclusively at Royal Alexandra Hospital, Edmonton, Alberta by 8 vitreoretinal surgeons.

Clinic charts of patients who underwent PPV for symptomatic floaters between 2017 and 2023 were reviewed. Eyes with a clinical diagnosis of PVD or asteroid hyalosis were included in the study. Standard 23-25G vitrectomies were performed via trans pars plana approach using Alcon Constellation System (Alcon, 6201 South Freeway, Fort Worth, TX 76134 − 2001, United States). Chromovitrectomy with dilute triamcinolone was utilized for assessment of a pre-existing PVD and to facilitate separation of an incomplete PVD. Anterior hyaloid was routinely disrupted in clear anterior vitreous floaters. Following vitreous removal, a 360 degree depressed exam was performed to look for peripheral retinal holes or tears. These were treated with retinopexy. Based on surgeon preference, the vitreous cavity was either left with infusion fluid or air tamponade. In cases with air tamponade, complete air insufflation of vitreous cavity was performed. Sclerotomies were inspected for leakage at the end of each case, and if suturing was required, 8 − 0 vicryl or 6 − 0 plain gut was used (Ethicon, Johnson & Johnson MedTech, 200 Whitehall Drive, Markham, ON L3R 0T5). Each case was concluded with subconjunctival dexamethasone and cefazolin (or tobramycin if there is an allergy to cefazolin). There was no intravitreal triamcinolone left at the end of the surgery and no subtenon triamcinolone was used at the end of the case. Post operative drops include moxifloxacin four times per day for two weeks and prednisolone acetate 1% four times per day for four weeks.

Only cases with symptomatic floaters due to PVD (as noted on examination and optical coherence tomography) and asteroid hyalosis were included. Those who had previous vitrectomies, previous scleral buckle surgery, secondary myodesopsiae (i.e. uveitis) and preoperative vitreous hemorrhage were excluded.

Chart review was conducted to extract eligible patient’s age, gender, ocular laterality, preoperative lens status, if phacovitrectomy was performed, post-operative VA, postoperative complications and their timing as well as any patient remarks at post operative visits regarding their satisfaction with the surgery. Post-operative best corrected visual acuity (BCVA) was deemed as the visual acuity recorded at (i) 3 months; (ii) or their earliest appointment after 3 months; (iii) or their last appointment at Alberta Retina Consultants. VA was converted to logMAR equivalents as outlined by Lange et al. [13].

## Results

### Overview

A total of 452 eyes who underwent PPV for symptomatic floaters were identified. Following application of exclusion criteria, 42 patients were excluded leading to inclusion of 410 eyes of 308 patients.

Table 2 illustrates demographic data of the study. There were 157 male and 151 female patients with the average age of participants being 67.5 years (range 36–93 years). Of the total number of eyes, there were 215 right (52%) and 195 left (48%) eyes. Vitrectomy was performed due to symptomatic PVD in 400 eyes (98%) with 10 eyes (2%) being symptomatic due to asteroid hyalosis.

**Table 2:** Demographic information of eyes undergoing PPV for VDM

Demographic Information	*n*	%
Average Age ± SD	68 ± 9	
Age Range	36–93	
Total Number of Eyes	410	
**Patient Sex**		
Males	157	51
Females	151	49
Average Length of Follow-up (months)	12.5	
Length of Follow-up Range (months)	0–90	
**Ocular Laterality**		
Right	215	52
Left	195	48
**Lens Status**		
Phakic	181	44
Pseudophakic	229	56
**Type of Intraocular Lens**		
Multifocal Intraocular Lens	15	7
Monofocal Intraocular Lens	214	93
**Indication for Vitrectomy**		
Symptomatic Floaters Secondary to PVD	400	98
Asteroid Hyalosis	10	2
**Type of Surgical Procedure**		
Pars Plana Vitrectomy	308	75
Phacovitrectomy	102	25

Among the 410 eyes included, 181 eyes were phakic (44%) and 229 eyes were pseudophakic (56%). There were 308 pars plana vitrectomy (75%) procedures performed while 102 eyes (25%) underwent combined intraocular lens implant and pars plana vitrectomy. Of the pseudophakic eyes, 15 eyes (6.5%) had multifocal intraocular lenses (MFIOL) and 214 (93.4%) had monofocal intraocular lenses (MIOL). Of the phakic eyes, 102 eyes (56%) underwent combined phacoemulsification, intraocular lens implant and pars plana vitrectomy which left 79 (44%) eyes phakic after PPV.

### Visual acuity

Preoperative and postoperative visual acuities were extracted from each chart. The mean preoperative logMAR was 0.22 (range 0–0.23) and postoperative logMAR was 0.21 (range 0–0.23). An average relative change in logMAR of -0.01 was noted between preoperative and postoperative visual acuity.

### Patient satisfaction

**Fig. 1:**
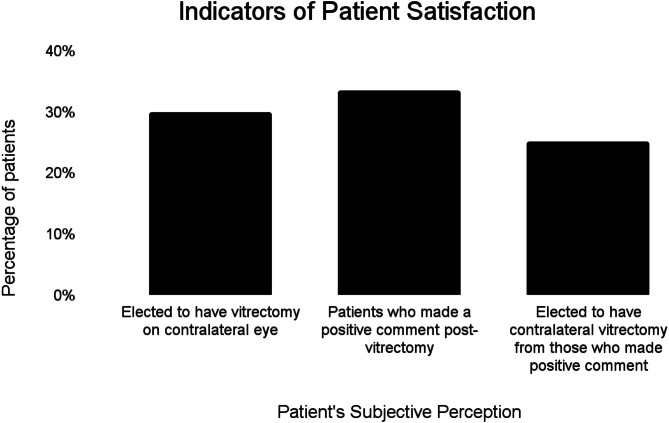
Indicators of patient satisfaction following vitrectomy for floaters/vision degrading myodesopsia

Patient charts were qualitatively reviewed for comments pertaining to overall patient satisfaction (Fig. 1). Comments that were regarded positive (i.e. happy with the surgery, resolved floaters since the surgery, expressive interest in second eye surgery) were included. One hundred and three patients (33.4%) were found to have made positive comments. Of these patients, there were 26 patients (25.2%) that elected to undergo floaterectomy in the contralateral eye. There was no formal questionnaire about patient satisfaction that was conducted at the follow-up visits; therefore, some charts contained no comments and the decision to enter any comments made by the patient was dependent on the ophthalmic technician that was screening the patient at the time. Another measure of patient satisfaction was the decision to pursue PPV for floaters in contralateral eye after undergoing PPV for floaters in one eye. Of the total 308 patients who underwent PPV for symptomatic floaters, 92 patients (29.9%) elected to undergo floaterectomy for contralateral eye.

### Repeat vitrectomy for persistent floaters

Three eyes (0.73%) elected to undergo repeat vitrectomies for unresolved symptomatic floaters. Only one of the repeat vitrectomies were performed under 6 months after initial PPV with remaining two vitrectomies being done more than 6 months postoperatively. Average time between initial and repeat vitrectomies was 16.7 months.

### Complications

#### Overview of complications

The following were considered postoperative complications: retinal detachment, vitreous hemorrhage, endophthalmitis, cataract progression, dislocated intraocular lens, elevated intraocular pressure, cystoid macular edema, macular hole, epiretinal membrane.

**Fig. 2:**
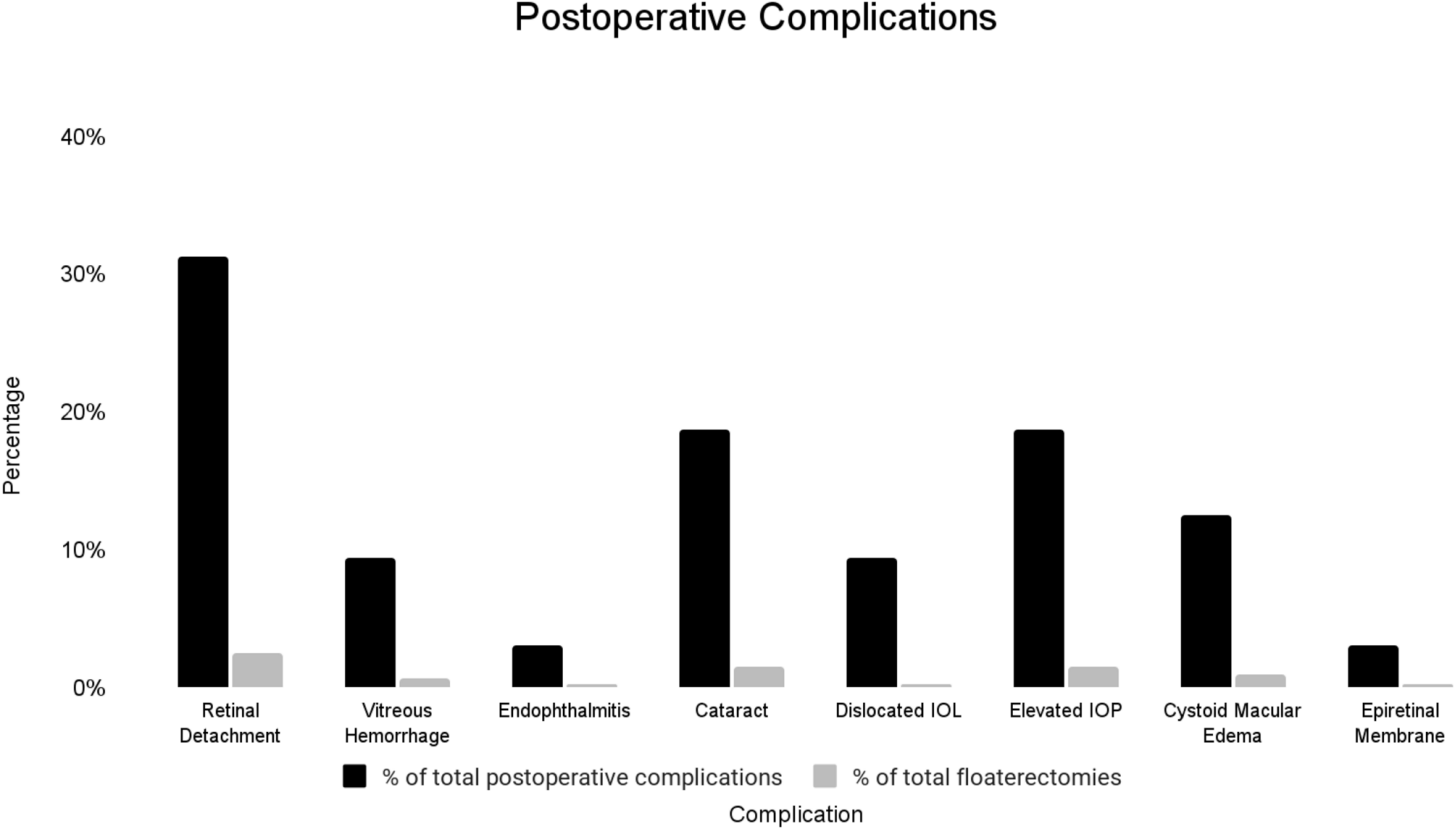
Rates of postoperative complications and proportionality of complications

Of the 410 eyes included in the study, 30 eyes (7.3%) had postoperative complications (Fig. 2). The 32 eyes accounted for 32 cases of postoperative complications (7.8%) with 4 eyes in the group having two different complications each.

#### Retinal detachment

Retinal detachments (RD) occurred in 10 eyes (2.4%). Five eyes (1.2%) developed retinal detachments in under 3 months following the procedure while the remaining 5 detachments occurred more than 6 months following surgery. Seven of these eyes were pseudophakic prior to vitrectomy and three eyes were phakic with only one of the phakic eyes undergoing combined phacovitrectomy. Average age of those who had retinal detachments was 65 years. Among the eyes with RDs, three eyes were highly myopic and one eye had a history of contralateral RD. The average postoperative VA for eyes that had RDs was logMAR 0.16; there was one outlier with 20/400 vision on Snellen visual acuity with the remaining VA ranging from 20/20 to 20/40. The eye with the final visual acuity of 20/400 had multiple complications including a vitreous hemorrhage at 1 month postoperative period after RD repair and a second RD at 1.5 months post-operative period which required surgical management. Intraoperatively during silicone oil removal at 7 months after RD repair, a macular hole was also noted.

#### Elevated intraocular pressure

Six eyes (1.5%) developed intraocular hypertension (IOP > 21) following surgery with a mean IOP at two weeks post-operative status of 34 ± 5.18 mmHg. Four eyes with elevated IOP resolved with topical medical therapy in under 3 months, one eye’s IOP resolved spontaneously following cessation of steroid topical therapy and one patient was lost to follow-up following commencement of IOP lowering therapy. There were no cases of postoperative ocular hypotony. None of the patients required a surgical intervention to reduce intraocular pressure.

#### Cataract progression

Of the phakic eyes, 102 eyes (56%) underwent combined phacoemulsification, intraocular lens implant and pars plana vitrectomy which left 79 (44%) eyes phakic after PPV. Postoperative cataract progression requiring surgery occurred in six eyes (1.5%). Four eyes had mild cataracts noted prior to vitrectomy. Two eyes had cataractogenesis within 3 months and two eyes between 3 and 6 months. Two eyes developed cataracts after 6 months. Overall, the mean time from initial vitrectomy to the diagnosis of a visually significant cataract requiring surgery was 4.75 months.

#### Cystoid macular edema

Four eyes (0.98%) developed postoperative cystoid macular edema (CME). In two of these cases, patients had undergone phacovitrectomy. The other two patients were noted to be highly myopic. All patients were started on topical therapy, in which 3 cases resolved within 1 to 2 months and the remaining case was lost to follow-up. The average VA for eye that developed CME was logMAR 0.075 with VA ranging from 20/20 to 20/30.

#### Vitreous hemorrhage

Three eyes (0.73%) had vitreous hemorrhages (VH) within 3 months of surgery, all of which required surgery for management. The average VA for eyes who had postoperative VH was logMAR 0.67 with Snellen visual acuity including 20/40, 20/50 and 20/400. The eye with VA of 20/400 developed an RD one month after surgery for VH and another RD and proliferative vitreoretinopathy (PVR) within a month of the first RD surgery. Other factors that contributed to the overall poor visual acuity include plaquenil retinopathy and proliferative diabetic retinopathy (PDR).

#### Dislocated intraocular Lens

Dislocation of the intraocular lens (IOL) occurred in 1 eye following vitrectomy (0.24%) which occurred 3 years after surgery. It was surgically managed with an insertion of Akreos IOL.

#### Endophthalmitis

There was only one case of endophthalmitis (0.24%) at 3 days following vitrectomy. The final VA for this eye was 20/60 (20/40 preoperatively). Other than recent surgery, there were no other risk factors identified for development of endophthalmitis.

#### Epiretinal membrane

There was one eye with a new epiretinal membrane (ERM) (0.24%) which was noted at 1 month postoperatively.

#### Subanalysis: combined phacovitrecomy cases

Of the 181 phakic eyes included in the study, 102 eyes (56%) underwent combined phacoemulsification, intraocular lens implant and pars plana vitrectomy. Among these cases, 7 eyes had postoperative complications, thereby contributing to 19% of the total cases of complications. These included 2 eyes with CME, 3 eyes with RD and 2 eyes with postoperative elevated IOP.

## Discussion

Vitreous floaters are a common finding on clinical examination that are burdensome for those with VDM. Currently available options for VDM management include observation, laser vitreolysis or pars plana vitrectomy. Numerous studies have found that pars plana vitrectomy is effective at reducing or resolving floater-associated symptoms with high patient satisfaction, however, complications have been noted [10]. These complications are especially important as symptomatic floaters are not regarded as an acute, vision threatening disease requiring immediate intervention deeming PPV for floaters to be an elective procedure. Many patients evaluated for possible vitrectomies for floaters have a normal or near-normal baseline visual acuity. This study aimed to demonstrate the effectiveness of PPV for VDM within a large sample size from a subset of the Canadian population.

The current study demonstrated a mean preoperative BCVA expressed as logarithm of the mean angle of resolution (logMAR) of 0.22 (20/100 Snellen equivalent) and mean postoperative logMAR of 0.21 (20/280 Snellen equivalent) with no statistically significant difference (*p* = 0.67). However, existing literature supports improved BCVA following floaterectomy. A systematic review with meta analysis by Dysager et al. reports improvement of BCVA by 0.08 logMAR [10]. In their retrospective studies, Khan et al. found that mean logMAR improved from 0.72 +/- 0.62 preoperatively to 0.4+/- 0.55 postoperatively (*P* < 0.001) [14] while Mason et al. reported statistically significant improvement from mean Snellen visual acuity of 20/40 preoperatively to 20/25 postoperatively (*P* < 0.0001) [11]. A possible explanation for the discrepancy in our data is the existence of outliers of preoperative BCVA in the study population; 18 eyes had a preoperative visual acuity of 20/100 or worse with one patient having hand motion vision. Furthermore, postoperative BCVA were measured at various points in the postoperative course with measurements made either at (i) 3 months; (ii) or their earliest appointment after 3 months; (iii) or their last appointment at Alberta Retina Consultants. Consequently, those who underwent an uneventful PPV for symptomatic floaters and were discharged back to their referring ophthalmologist or optometrist, their last known visual acuity at Alberta Retina Consultants was measured early on in their postoperative course when poor visual acuity is likely to be seen, which begs the question if the charts had the true postoperative BCVA.

Current literature demonstrates high patient satisfaction for PPV for VDM; some of the reported patient satisfaction rates with surgical outcomes include 88% [12], 85% [15], 96% [11]. In accordance with current literature, our study supports patient satisfaction with PPV for symptomatic floaters in two different ways. Of the total cases, 103 patients made positive comments postoperatively and 92 patients elected to undergo floaterectomy for contralateral eye. The discrepancy between the number of patients who made positive comments and decided to undergo contralateral floaterectomy (26 patients) and the number of patients who underwent floaterectomy but did not make a positive comment (66 patients) is explained by the fact the study did not involve a patient satisfaction survey that was required to be filled about each patient nor involve each screening technician to document any comments made by the patients. The findings were based on a retrospective review of comments and the decision to document any patient comments was dependent on the screening technician. True rates of patient satisfaction require a dedicated questionnaire as well as documentation of the patient’s lens status and if they are pseudophakic, it will be important to make note if they have an MIOL or a MFIOL. Studies that have quantified symptoms with contrast sensitivity function (CSF have also reported improvement in CSF with vitrectomy [5].

In this study, 25% of the eyes underwent phaco-vitrectomy while 75% underwent PPV alone. Of the combined cases, 7 eyes accounted for 19% of the total cases of postoperative complications. Current literature lacks studies that compare combined phaco-vitrectomy and PPV for VDM with regards to their visual outcomes and postoperative complications. However, many studies have explored this comparison in the setting of ERM [16], rhegmatogenous retinal detachment [17] and full thickness macular hole [18] and there have not been significant differences in safety and efficacy. Nevertheless, a cost advantage to performing phacovitrectomy for patients with an indication for vitrectomy and a visually significant cataract in comparison to sequential PPV and cataract surgery is observed [19].

MFIOLs are known to be associated with poor CSF [20, 21] which is also seen with VDM, thus our initial postulation was to observe more patients with MFIOLs electing to undergo PPV for VDM. Among the pseudophakic eyes in the current study, 7% had MFIOLs while 93% had MIOLs. In a prospective study by Nguyen et al., they observed that CSF was 25% worse in MFIOL than in MIOL [22]. As MFIOLs gain popularity among patients, the number of patients with MFIOL that undergo PPV for VDM may increase which would create grounds for further studies that explore lens status on VDM.

The total complication rate was 7.3% accounting for 32 complications in 30 eyes out of the total 410 eyes that underwent PPV for VDM. The most common postoperative complications were retinal detachment, cataract formation and elevated IOP while cases of endophthalmitis, dislocated intraocular lens, and epiretinal membrane were among the rarest of complications. There was a statistically significant difference in the average preoperative BCVA logMAR of 0.15 to postoperative BCVA logMAR of 0.3 (*P* = 0.04), which leads to the extrapolation of significant impact on BCVA for those with postoperative complications following PPV for VDM.

This study found the rate of postoperative RDs following PPV for VDM to be 2.4%, which accounted for 25% of total postoperative complications. Current literature reports the incidence of RD following vitrectomy for floaters to vary between 0 and 10.9%, which deems the rate of 2.4% in this study to be at the lower end reported in literature [7, 10–12, 23–47]. Among the eyes with RDs, 40% had additional risk factors for RD including high myopia and history of contralateral RD. All but one of the cases had good final visual acuity outcomes (Snellen visual acuity 20/20 to 20/40).

Cataract progression is a known clinical sequelae of vitrectomy. The study found six cases (1.5%) of clinical significant cataracts requiring cataract surgery within the follow up period of the study. These six cases were among the 79 eyes who were left phakic after pars plana vitrectomy. The cataract progression was noted to have occurred at various time points in the postoperative course (3 cases within 3 months; 2 cases between 3 and 6 months and one case at 8 months). Two cases had pre-existing cataract noted prior to PPV. Overall, the mean time from initial vitrectomy to the diagnosis of a visually significant cataract requiring surgery was 4.2 months. Reported rates of cataract progression have been variable in current literature with some cases having pre-existing cataracts [26] similar to this study. Lin et al. [26] and Waseem et al. [39] found no clinically significant cataracts postoperatively. Whereas, Zeydanli et al. reports 48.6% developed cataracts at median of 16 months after floaterectomy [40]. Of note, the average length of postoperative follow up for the patients included in the current study was 12.5 months. Therefore, those who had cataract progression following discharge from the clinic were not included. This would have artificially led to a lower rate of cataract progression found in the study that is not reflective of the true rate of cataract progression after floaterectomy. It could have been addressed with longer follow up time.

In studies that reported the rate of endophthalmitis following vitrectomy for floaters, rates ranged from 0 to 2.1% [11, 24–29, 31–47]. In comparison to other ophthalmic surgeries, vitrectomies have low rates of endophthalmitis occurring in about 0.046% of patients [48]. In the current study, there was only one case (0.24%) endophthalmitis with final visual acuity of 20/60 (20/40 preoperatively).

There were three cases (0.73%) of postoperative vitreous hemorrhage, all of which occurred within 3 months of surgery. Current literature reported postoperative vitreous hemorrhage rate ranging from 0 to 5.13% [11, 15, 23, 25, 26, 27, 31, 35, 36, 37, 38, 39, 40, 26, 42, 43, 45–46]. All eyes required a second vitrectomy to clear the vitreous hemorrhage with one patient returning to their baseline VA of 20/40 before their floaterectomy. The second patient’s VA decreased significantly following vitrectomy for VH with an RD that developed within the first month after surgery, and another RD secondary to PVR that developed within two months. Additional factors include plaquenil retinopathy and PDR. Compared to the baseline VA of 20/30, final VA was 20/400 following multiple vitrectomies and silicone oil removal. The last patient was lost to follow-up following vitrectomy for vitreous hemorrhage. Two of the three cases had additional risk factors of hypertension with one case having a history of diabetes. None were found to be on anticoagulants.

Rates of post-vitrectomy CME following floaterectomy are reported to be between 0 and 20% with the current study reporting a rate of 0.98% [12–13, 15, 26, 28–29, 31, 33, 35–36, 40, 43, 44, 45, 46, 47]. A study by Mossa et al. [29] reported a 20% rate of CME, which occurred in 2 eyes of 1 patient in a sample of 10 eyes. In the current study, all cases of CME resolved with topical medical therapy. Three of these cases occurred within 3 months of operation, whereas 1 case of CME occurred 4 years after vitrectomy. 50% of the cases that developed CME had combined phacoemulsification and vitrectomy procedures. Phacovitrectomy has been found to have higher rates of CME compared to vitrectomies alone. For example, Park et al. found rates of increased macular thickness to be 15.4% after phacovitrectomy for ERM versus 4.8% following PPV alone in pseudophakic eyes [49]. This may be a possible explanation for the higher rate of CME observed in the current study in comparison to a sample that underwent PPV alone.

To our knowledge, no study has reported cases of dislocated IOL following PPV for VDM. The current study, there was only one case of postoperative dislocated IOL which required second surgery with Akreos posterior chamber intraocular lenses (Akreos Advanced Optics Intraocular Lenses; Bausch and Lomb, 400 Somerset Corporate Blvd, Bridgewater, NJ 08807).

There was one case of postoperative ERM after vitrectomy. This case had prior cataract extraction with IOL insertion which has been found to have an increased likelihood of anatomical progression (worsening traction or increased macular thickness) or functional visual decrease (visual acuity decrease or worsening metamorphopsia) [50].

Three pseudophakic cases (0.73%) elected for repeat PPV due to persistent, visually significant floaters despite having primary floaterectomy. Studies have reported rates of 1.3% and 5.1% of patients electing for repeat vitrectomies to ameliorate persistent symptomatic floaters [23, 51]. One explanation for this persisting symptomatology is due to the lack of anterior vitreous clearance in its propinquity to the nodal point of the eye. This can be particularly noticeable in individuals with MFIOL due diffractive scatter that occurs in the retrocapsular region. Unlike floaters generated from a posteriorly positioned PVD (e.g. Weiss ring) which can create an umbral effect, the anteriorized floaters obstruct the passage of light by antumbral and penumbral effects [37].

Rubino et al. conducted a retrospective cohort study of cases that return to the operating room after pars plana vitrectomy for vitreous opacities [53]. Among patients in the IRIS registry, 2187 eyes or 12.4% were found to have returned to the operative room for cataract surgery and 3.7% returned for non-cataract procedures, of which 2.6% were for retinal detachment repair. In the current study, there was a total of 24 cases or 5.8% of total floaterectomies which return to the operative room after surgery; of these, there was 3 cases of repeat vitrectomy for non-resolving floaters (0.73%), 10 retinal detachments (2.4%), 6 cases of cataracts that required surgery (1.5%), 3 vitreous hemorrhages (0.73%), 1 case each of dislocated IOL (0.24%) and endophthalmitis (0.24%). The rate of retina detachments requiring surgical repair in the current study was comparable to findings of the IRIS registry. As discussed earlier, the rate of cases requiring cataract surgery may be artificially low given that many patients were discharged back to their primary ophthalmologist or optometrist and thereby, any cases of cataract progression after discharge were not noted.

A strength of the current study is its large sample size spanning seven years. Given the long term follow up of patients at this tertiary care center, any postoperative complications occurring years after the procedure were able to be included. In addition, our sample consisted of a relatively equal number of male and female patients from a wide range in age, eliminating any potential statistical bias due to gender and age.

Limitations of our study of this research related to the retrospective nature of the study. There were no strict inclusion criteria as utilized by other studies such as duration of symptoms or confirmation of vitreous floaters by imaging modalities such as ultrasonography [25, 27]. Further, findings pertaining to patient satisfaction were drawn from comments made at visits wherein no dedicated standardized patient satisfaction questionnaire was completed, but rather was dependent on documentation preferences of various providers. This warrants further investigation to corroborate the findings of the study in a prospective, multicentered analysis.

## Conclusion

Our study supports pars plana vitrectomy as an effective option for management of symptomatic floaters. Our observed complication rate was 7.3% which included adverse events occurring up to 3 years after the procedure. There was no significant difference in visual acuity noted in the preoperative and postoperative period with a relative improvement of logMAR of -0.01. A statistically significant difference in these measures were noted in the eyes with complications, yielding the possibility of significant impact on visual acuity in the event of a postoperative complication.

## Data Availability

The datasets used during the study are available from the corresponding author on reasonable request as there are restrictions that apply in order to protect patient privacy. The data will be available upon request and with permission from Alberta Research Information Services (ARISE) System through the University of Alberta (ID: Pro00141654).

## Abbreviations

PVD: Posterior vitreous detachment
VDM: Vision degrading myodesopsiae
VA: Visual acuity
CSF: Contrast sensitivity function
PPV: Pars plana vitrectomy
BCVA: Best corrected visual acuity
MFIOL: Multifocal intraocular lenses
MIOL: Monofocal intraocular lenses
LogMAR: Logarithm of the minimum angle of resolution
RD: Retinal detachment
IOP: Intraocular pressure
CME: Cystoid macular edema
VH: Vitreous hemorrhage
PVR: Proliferative vitreoretinopathy
PDR: Proliferative diabetic retinopathy
IOL: Intraocular lenses
ERM: Epiretinal membrane
